# Bioceramics/Electrospun Polymeric Nanofibrous and Carbon Nanofibrous Scaffolds for Bone Tissue Engineering Applications

**DOI:** 10.3390/ma16072799

**Published:** 2023-03-31

**Authors:** Zahra Ebrahimvand Dibazar, Lei Nie, Mehdi Azizi, Houra Nekounam, Masoud Hamidi, Amin Shavandi, Zhila Izadi, Cédric Delattre

**Affiliations:** 1 Department of Oral and Maxillo Facial Medicine, Faculty of Dentistry, Tabriz Azad University of Medical Sciences, Tabriz 5165687386, Iran; 2 College of Life Sciences, Xinyang Normal University, Xinyang 464000, China; 3 Department of Tissue Engineering and Biomaterials, School of Advanced Medical Sciences and Technologies, Hamadan University of Medical Sciences, Hamadan 6517838636, Iran; 4 Department of Medical Nanotechnology, School of Advanced Technologies in Medicine, Tehran University of Medical Sciences, Tehran 1416634793, Iran; 5 Université Libre de Bruxelles (ULB), École Polytechnique de Bruxelles, 3BIO-BioMatter, Avenue F.D. Roosevelt, 50-CP 165/61, 1050 Brussels, Belgium; 6 Pharmaceutical Sciences Research Center, Health Institute, Kermanshah University of Medical Sciences, Kermanshah 6714869914, Iran; 7 USERN Office, Kermanshah University of Medical Sciences, Kermanshah 6714869914, Iran; 8 Clermont Auvergne INP, CNRS, Institut Pascal, Université Clermont Auvergne, F-63000 Clermont-Ferrand, France; 9 Institut Universitaire de France (IUF), 1 Rue Descartes, 75005 Paris, France

**Keywords:** bone tissue engineering, nanofibers, mineralization, electrospinning

## Abstract

Bone tissue engineering integrates biomaterials, cells, and bioactive agents to propose sophisticated treatment options over conventional choices. Scaffolds have central roles in this scenario, and precisely designed and fabricated structures with the highest similarity to bone tissue have shown promising outcomes. On the other hand, using nanotechnology and nanomaterials as the enabling options confers fascinating properties to the scaffolds, such as precisely tailoring the physicochemical features and better interactions with cells and surrounding tissues. Among different nanomaterials, polymeric nanofibers and carbon nanofibers have attracted significant attention due to their similarity to bone extracellular matrix (ECM) and high surface-to-volume ratio. Moreover, bone ECM is a biocomposite of collagen fibers and hydroxyapatite crystals; accordingly, researchers have tried to mimic this biocomposite using the mineralization of various polymeric and carbon nanofibers and have shown that the mineralized nanofibers are promising structures to augment the bone healing process in the tissue engineering scenario. In this paper, we reviewed the bone structure, bone defects/fracture healing process, and various structures/cells/growth factors applicable to bone tissue engineering applications. Then, we highlighted the mineralized polymeric and carbon nanofibers and their fabrication methods.

## 1. Introduction

Bone defects are a high prevalence and devastating injuries, generally caused by trauma, debridement for bone tumors, infection, or nonunion (a bone that is not healing). The effective treatment options include bone transport distraction osteogenesis, shortening the bone, and bone grafting. Despite their efficacy, they have substantial drawbacks and could not be considered as a completely effective treatment. Accordingly, researchers have been seeking alternatives to circumvent the shortcomings of conventional treatments for large bone defects [1,2,3]. Tissue engineering is an emerging concept aiming to combine engineering, cells, biomaterial concepts, and proper biochemical and physicochemical factors to repair, replace, or improve damaged tissues [4,5,6]. Bone tissue engineering has attracted enormous attention during the last few years due to promising results obtained using this approach. Scaffolds play a central role in the bone tissue engineering approach as the supports and modulator for cell attachment, proliferation, and differentiation, and as the carrier for osteogenic substances. It is critical to point out that the scaffold’s morphology, microstructure, porosity, mechanical, and physicochemical characteristics must be similar to the natural bone as much as possible [7,8,9].

A wide range of materials such as strontium- and cobalt-substituted bioactive glasses, hydroxyapatite/tricalcium phosphate (HA/TCP), gelfoam surgical sponges, and nanofibers have been investigated for bone tissue engineering applications [10,11]. Among them, nanofibrous structures have shown fascinating performance and attracted considerable attention. Nanofibers are 2D structures with a high surface-to-volume ratio that mimic the extracellular matrix (ECM) of bone. Moreover, it is possible to prepare 3D constructs using nanofibers, applicable for bone tissue engineering applications [12,13,14]. Different methods have been developed to fabricate nanofibers, such as molecular self-assembly, template synthesis, temperature-induced phase separation, drawing, and electrospinning, which have their pros and cons. Electrospinning is a sophisticated and straightforward method for preparing nanofibrous structures with various morphology, structure, porosity, and geometry [15,16,17,18].

Bone is a nanocomposite structure composed of collagen nanofibers, the organic phase of bone, and mineralized with hydroxyapatite crystals, the bone’s inorganic phase. Accordingly, researchers have proposed that composite constructs made from artificial nanofibers mineralized with bioceramics crystals would be a proper scaffold mimicking the native structure of bone. Hence, various polymeric nanofibers such as carbon nanofibers (CNFs) have been fabricated and composited with different bioceramics types to develop bone healing materials [19,20,21].

Because of their biocompatibility, ease of manufacture, and good electrical conductivity, CNFs are frequently employed in bone tissue engineering applications. This is because they provide the most favorable microenvironment for osteoblasts to proliferate and speed up the regeneration process [22,23]. Many applications, such as selective adsorption, polymer reinforcement, electrochemical catalysis, and hydrogen storage, are made possible by the special features of CNFs [23]. For the purpose of bone tissue engineering, a study by El-Aziz et al. in 2017 focused on the creation of carbon nanofiber sheets functionalized with hydroxyapatite (HA) and BSA. The biocompatible functionalized sheets that were altered with HA or HA and BSA were successfully prepared. For a period of three weeks, the functionalized sheets containing both HA and BSA were more biocompatible and contained less inflammatory cells (neutrophils and lymphocytes) than those with simply HA [24]. Aoki et al. discussed the application of electrospun CNFs in bone regeneration in their 2020 study. These nanofibers were created by electrospinning polyacrylonitrile, then thermally treating them at 1000 °C while being supplied with argon gas to create a thin, three-dimensional matrix. The trials were conducted utilizing rhBMP-2, an anabolic signaling molecule also utilized to treat numerous bone diseases, and the results demonstrated subsequent bone development [25].

This paper aims to discuss various types of nanofibrous scaffolds mineralized with different bioceramics by distinct methods and applied for bone tissue engineering. First, we introduce the structure and mechanical properties of bone and its healing process, and then discuss the basic principles of bone tissue engineering and nanofibers’ applications in bone tissue engineering. Next, we review the mineralized nanofibrous nanocomposites applied as the tissue engineering applications. Finally, the challenges, solutions, and prospective are presented. Although there are some review papers on the application of nanofibers in bone tissue engineering, there is room for more insight, especially on the combination of nanofibers’ technology with bioceramics chemistry.

## 2. Hierarchical Structure of Bone

Bone is a heterogeneous and anisotropic bio-composite structure that supports and protects the body. The multi-scale structural geometry that organizes this structure into hierarchical levels affects how well it can carry out functions [26,27]. It is known today that bone tissue structure can be classified into five structural levels, from macro- to nanocomponents [26,28,29]

The macroscopic or organ level actually forms the anatomical shape of the bone tissue and includes the outer geometry and internal architecture of the trabecular and cortical bone, which affect the mechanical properties of this tissue. At the next structure level, which defines the micro- and sub-microstructures, the osteons, lamellae and single trabecula structures can be seen, which actually create the extracellular matrix (ECM) of bone. The osteons are composed of concentrated lamellae of mineralized collagen fibrils. This level has a highly porous structure and is surrounded by small cavities containing blood vessels and nerves. The porosity and channels are very important for cell migration and vascularization in the bone. On the lowest level of structural bone, nano- and sub-nanostructures represent the components of the nanocomposite materials. The mineralized collagen fibrils that are the main building block of all types of bone have a diameter of about 0.1 μm. Collagen is made by osteoblasts, which are bone-forming cells that then secrete collagen molecules into the extracellular space, which later forms into a fibril structure [26,27,28,29,30,31,32].

**Figure 1:**
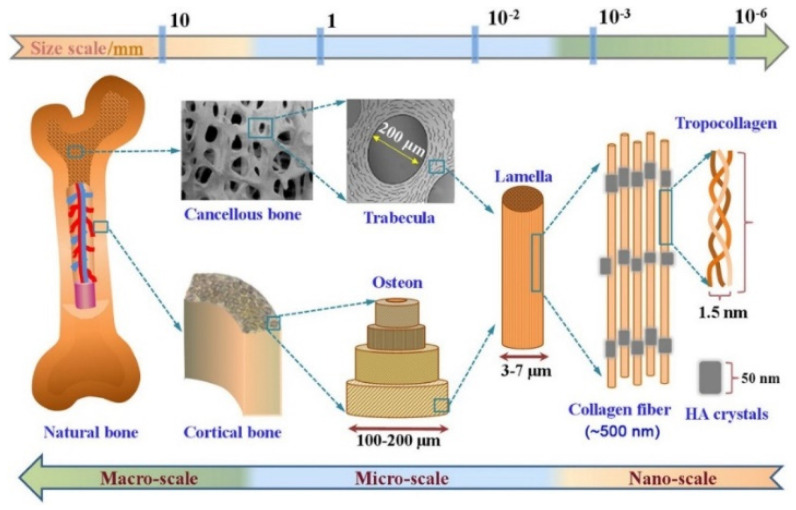
The hierarchical multi-scale structure of natural bone ranging from the nanoscale to macroscale. Reproduced with permission from Ref. [38].

The collagen type 1 molecule is a large protein in a bone organic matrix and the mineralized fibrils are based on parallel collagen molecules reinforced with thin calcium phosphate mineral platelets. The bone mineral has a non-static moiety due to poorly crystalline hydroxyapatite, so calcium (Ca^2+^) and phosphate ions (Pi) can be easily substituted [33,34,35]. On the other hand, the non-collagenous organic proteins provide natural bone with its remarkable mechanical properties. Osteons in both microscopic and molecular levels create the extracellular matrix (ECM) of bone, whose organic matrix primarily contains collagen (Type I) [36,37]. Overlaid on this, each of these scales or hierarchical levels, molecular (e.g., collagen type and hydroxyapatite (HAP) stoichiometry) (Figure 1) and microscopic (e.g., microstructure and porosity), will have an influence on the biomechanical characteristics of bone and cell behavior [38,39].

## 3. Mechanical Properties of Bone

The bones in the human skeleton have a variety of functions; hence, they are certainly not mechanically identical. The mechanical properties of bone are essential for the ability of skeletons to support the movement and protect the vital organs of the body [40,41]. In fact, bones represent a system with a complex function and structure. The biomechanical properties of bone tissue are affected by the physiological and metabolic conditions of the body. In addition, there are tissues such as fat, blood vessels, cartilage, and nerves in this tissue. In general, the bone structure has a decisive influence on its mechanical properties. As we presented, bone is a biological composite material that is made of two major components: organic and inorganic; organic components (ossein protein and collagen fibers) are responsible for flexibility and elasticity, and the hardness of the bone tissue is due to the inorganic part (phosphoric and carbonic acids) [41,42,43,44,45].

Bone is a heterogeneous composite material with a mineral phase, hydroxyapatite (Ca_10_(PO_4_)_6_(OH)_2_), an organic phase (90% type I collagen, 5% noncollagenous proteins (NCPs), 2% lipids by weight), and water as its least abundant components. There are two main types of proteins found in the bone extracellular matrix: (a) structural proteins (such as collagen and fibronectin) and (b) proteins with specialized functions, such as those that (i) control the diameter of collagen fibrils, (ii) act as signaling molecules, (iii) act as growth factors, (iv) act as enzymes, and (v) have other functions.

Previous studies based on mechanical models have shown the important mechanical properties of bone and the role of mineral platelets in the strengthening of bone material [42,43,46].

At the molecular scale, the interaction between collagen molecules and inorganic hydroxyapatite (HAP) as well as the amount of minerals have been identified to play an important role in providing bone strength and toughness [46,47,48]. For this, an important characteristic of this material is the mechanical properties of bone. Because of the complex composite structure, bone has excellent mechanical properties for its functions that affect load-bearing capacity. Bone properties depend on the constituent properties and the geometrical and topological characteristics of the complex. For these reasons, understanding how bone components are arranged is important to the successful engineering of this tissue. Therefore, the analysis of the mechanical properties of the bones should be able to characterize the geometric and topological relationships between all components [49,50].

Besides the effects of the amounts of cortical and trabecular bone on whole-bone mechanical properties, structural properties, and geometries, such as bone shape, bone size, cortical bone thickness, and the cross-sectional spatial distribution of trabecular bone, are key factors that change with aging and disease. However, research has shown that the amount of minerals in the bone changes little with aging, and this trend is also reflected in their stiffness [41,51]. Therefore, the study of the mechanical behavior of whole bones can be more complex than the study of cortical or trabecular bone. It can be said that the mechanical properties of bone are intermediate between type I collagen and HAP [52]. HAP crystals are arranged in the length of collagen fibrils. Thus, this structure combines the mechanical advantages of both materials. HAP provides structural rigidity and compression strength, while the more flexible collagen fibers prevent brittle cracking and provide improved tensile properties. Interestingly, the bone is a very weak organ against tension but is strongest in stress tolerance [52,53,54].

The ideal mechanical characteristics of bone are the outcome of its complex hierarchical structure from a nano- to macro-surface, which is described further. In general, the mechanical properties of the trabecular bone are due to the high-porosity structure and the mechanical characteristics of a single trabecular bone. Bone porosity is also arranged hierarchically, and this porosity has a significant effect on bone mechanical properties [39,55]. As the basic block of bone is the mineralized collagen fibril, more attention has been paid to understand its mechanical function. The organic matrix is under pressure because of how the mineral particles react to the pressure [56,57]. In a mechanical model, it was shown that the reaction of the inorganic particles and the organic matrix is such that under tensile loading, these particles bear the load, and the role of the organic matrix is to carry the charge between the particles through the shear [58].

Hence, in order to compensate for the high elasticity of the organic elements, the shape of mineral particles is relatively large in size such that, at this scale, the sensitivity of these particles to flow decreases, which could be an advantage. Generally, it can be expected that the degree of stiffness and hardness of the whole structure can be adjusted by the number of mineral platelets in the elastic collagen fiber. Increased bone mineral density (BMD) increases bone hardness and reduces its fragility [59]. Overall, extensive efforts have been made to elucidate the structure of bone nanocomposite materials, their relationship to the mechanical properties, and the mechanism of deformation in the mineral tissue of this organ [60].

## 4. Bone Healing Process

Bone is a living organ and can grow; it can also regenerate itself and heal in case of injury. Bone can be defined as a unique tissue and, due to the importance of this organ in the stability of the whole body, its healing is very important. On the other side, the body can facilitate the repair or healing of bone. It is known that bone is one of a few tissues that can heal without forming a fibrous scar [61,62]. In this way, the process of bone healing recapitulates bone regrowth and can be considered a kind of tissue regeneration. However, bone healing is a dynamic biological process that results from the balance between mechanical and physiological stimuli [63,64,65,66].

In the classic histological terms, healing has been divided into two types including direct and indirect healing models [67]. The most common form of bone repair is indirect fracture healing, which involves multiple stages. The induction of hematoma and inflammation is the first stage at the time of bone fracture and can last for about 5 days. The organization of hematoma in an injured site that contains blood cells, mesenchymal stem cells, fibroblasts, osteoclasts, osteoblasts, cytokines, growth factors, and other hormones is an immediate response to fracture. The inflammatory cells of the acute immune system migrate to the fracture site and activate the upregulation of angiogenic factors to support the vascularization of the injured site. The inflammatory response, around 24 h after the fracture, is then balanced by the release of anti-inflammatory cytokines involved in the recruitment of cells to begin tissue healing. If the level of the acute inflammatory response is not reduced by the interaction of the immune system (for example, due to bacterial infection at the site of injury or chronic inflammatory disease), the healing process can be disrupted or inhibited and entered into the chronic phase [66,68,69,70].

In the next phase, the fracture healing process continues with the replacement of the gradual evolution of the hematoma into granular tissue, followed by a soft callus composed of fibrous tissue and cartilage. This stage, called the anabolic phase, is characterized by an increase in the volume of mesenchymal precursor cells. These cells are then differentiated into osteoblasts or cartilaginous cells for forming the extracellular matrix and cartilage. This process is induced by the replacement of the hematoma and the filling of the fracture gap [66,71,72,73]. Importantly, with the formation of soft callus tissue and the initial stability of the cells in the fracture area, the process of bone healing is not yet complete. Progressively, the process of endochondral ossification in soft callus tissue begins under mineralization and becomes a structure with a stronger mechanical property that is eventually replaced by tissue bone. In the final stage, which is the remodeling phase of the fracture area, the woven bone is slowly replaced by the lamellar bone. In this process, the remodeling is performed by the balance of callus absorption by osteoclasts and the deposition of the layered bone by the osteoblasts. Eventually, this process is completed by restoring the mechanical and biological functions of the bone [66,74,75].

**Figure 2:**
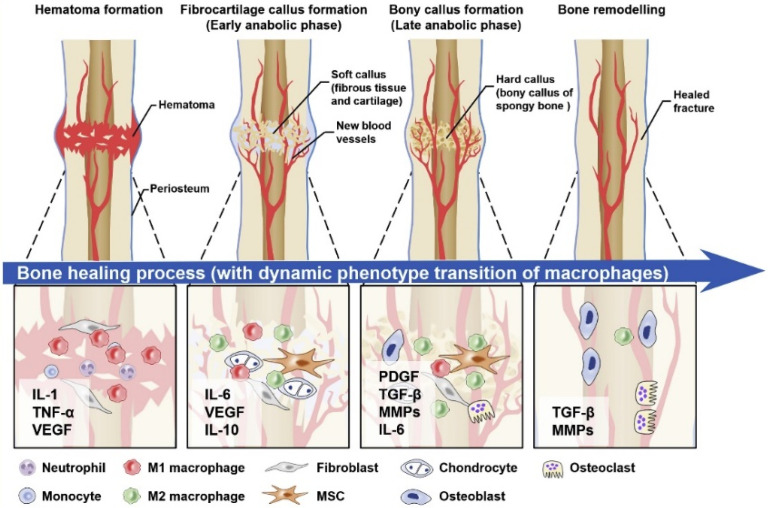
A graphical view of the direct bone healing process.

In contrast, the direct healing process (Figure 2), which is usually not a normal fracture repair process, aims to recover the correct fracture anatomy without creating a gap and is often needed after internal stabilization surgery. However, callus tissue does not form here and the two plates of bone at the site of fracture connect together, and the healing occurs to produce the bone by osteoclasts and osteoblast activities directly. The process of direct or primary, complete healing of the bones can take several months to several years, depending on the species [61,76,77].

## 5. Bone Tissue Engineering

Bone tissue engineering is a multidisciplinary approach aiming to accelerate bone regeneration. Bone tissue is considered the field of active interaction between biology and engineering. Tissue engineering and, specifically, bone tissue engineering combine cells, growth factors, and various biomaterials to provide a suitable microenvironment to regenerate tissue and its proper function. Scaffolds have a central role in bone tissue engineering application as the substrate to support and guide cell growth and as the vehicle for bioactive agents’ delivery. Various types of materials have been evaluated as the bone tissue engineering scaffold, such as synthetic and natural polymers, ceramics, and metals.

Due to the nature of each material, it has advantages or disadvantages, and the choice of a biomaterial is influenced by how closely the biomechanical qualities match those of the implantation location [78]. In addition, the optimal biomaterial needs to be osteoconductive in vivo, printable, biodegradable, and non-cytotoxic [2].

Biomaterials that are naturally derived (such as collagen, gelatin, chitosan, and starch) can provide cells with a variety of active stimuli and have positive characteristics such as biocompatibility and ECM resemblance [9,79], but even these could cause immunogenic problems, and controlling the biodegradability rate can be challenging [80]. Due to the greater control over the degradation rate and mechanical qualities, synthetic polymers, such as polylactic acid (PLA), polycaprolactone (PCL), and polyurethane (PU), are more frequently taken into consideration [7,81,82]. High strength and biocompatibility can be found in metals (such as titanium alloys) and inert ceramics (such as alumina and zirconia), but their usefulness in tissue engineering applications is limited due to their inability to degrade. It has been suggested to employ biodegradable metallic alloys, such as those based on magnesium, iron, and zinc, to get around this restriction [78,83]. Because of their resemblance to the mineral phase of bone, bioactive ceramics (such as hydroxyapatite (HA), tricalcium phosphate, and bioactive glasses) and their combinations have been used extensively. However, it should be noted that biological HA is non-stoichiometric and contains several impurities, most notably carbonates but also other anions and cations [84,85].

From a practical perspective, because of the unique properties of the biomaterial, scaffolds made from that material may have a number of disadvantages. The goal of composite biomaterials is to increase the processability, mechanical qualities, and bioactivity of the final scaffolds in order to better mimic certain traits of the target tissue (e.g., bone), as biological tissues can be thought of as natural composites [78,86]. So, in order to mimic the chemical makeup and mechanical characteristics of bone, composite bone scaffolds may blend inorganic compounds such as bioceramics with organic polymers, such as collagen [78].

## 6. Mineralized Nanofibers in Bone Tissue

Nanofibers are fascinating structures with a myriad of applications in various aspects of biomedicine. The similarity to ECM of tissues, the high surface-to-volume ratio, the ability to load multiple bioactive molecules onto/into nanofibers, and the availability of different fabrication methods have made nanofibers promising structures for tissue engineering applications. Electrospun nanofibers have found a critical role in bone tissue engineering as scaffolding materials. Electrospinning is a sophisticated, flexible, and straightforward technique capable of fabricating nanofibers from various natural, synthetic, and semisynthetic polymers with different geometry and conformation [17,22]. The critical challenge in bone tissue engineering is to fabricate scaffolds with the highest similarity to structure and mechanical properties of bone, which exhibit osteoconductivity and osteoinductivity. Nanofibers can be loaded with various osteogenic substances, such as bone morphogenetic proteins (BMPs). Moreover, it is shown that calcium phosphate bioceramics, such as hydroxyapatite (HA), bioglasses, β-tricalcium phosphate (β-TCP), and biphasic calcium phosphate, can confer osteoconductivity and osteoinductivity to the scaffolds [87,88]. CNFs are also thought of as reinforcing agents for polymers such as PCL, PLA, and others to improve or increase their mechanical, biocompatibility, and cellular responses and also mimic the nanoscale architecture of natural ECM [89,90]. Accordingly, mineralized nanofibrous composites are the main focus of bone tissue engineering. Electrospinning enables researchers to prepare mineralized nanofibrous composites with a high resemblance to bone structure. The mineralization of electrospun nanofibers can be conducted by various approaches, which have their advantages and disadvantages.

**Figure 3:**
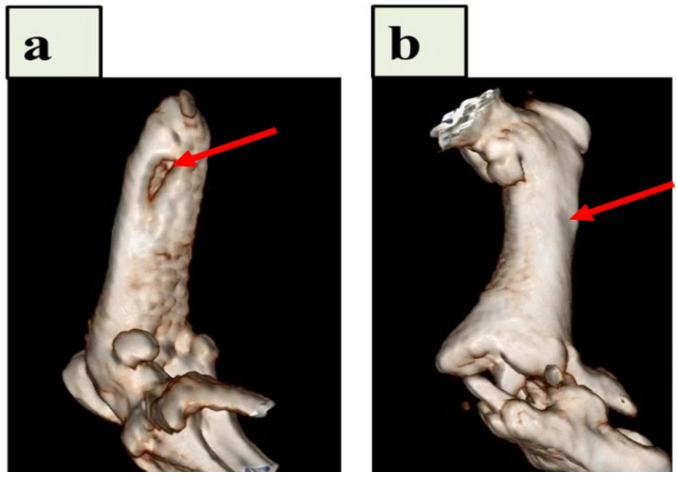
CT pictures of the implanted CNFs/HA nanocomposite that repaired the damaged femur in vivo. After 8 weeks following the accident, diagnostic 3D imaging (CT scan) was conducted of the femur bone defects. The arrow indicates the deficient area that was not healed in the control group (**a**) and the bone defect that was corrected as a result of the implanted CNFs/HA nanocomposite stimulating the growth of normal tissue (**b**). Reproduced with permission from Ref. [91].

Samadian et al. described the creation of hydroxyapatite (HA)-crystal-decorated osteoconductive electrospun CNFs for use as the scaffold for bone tissue engineering in an animal model. In this study, CNFs were produced by heating electrospun polyacrylonitrile (PAN) nanofibers, and a biomimetic method was used to mineralize the carbonized nanofibers. By considerably promoting in vivo bone formation in the rat femur defect site, the osteoconductive properties of the CNFs/HA nanocomposite were seen by computed tomography (CT) scan images and histological analysis (Figure 3). Additionally, the histomorphometric analysis revealed that the modified CNFs-treated group had the highest new bone production (61.3  ±  4.2%), which was significantly higher than that of the negative control group (the defect without treatment) (*p* < 0.05) [91].

Nekounam et al. used the electrospinning process and thermal treatments to create the various ECNFs/silica nanoparticles (ECNFs/SNPs) composites. SNPs were included into ECNFs to increase their hydrophilicity while lowering their electrical conductivity. Additionally, the inclusion of SNPs increased the biological activity of the ECNFs, including cell attachment, viability, and proliferation rate. It was determined that the enhanced MG-63 proliferation rate observed in the ECNFs/SNPs composite was caused by the composite’s potent osteoactive activity [22].

In another study, conductive scaffolds made of carbon nanofiber and gold nanoparticles (CNF/AuNP) were created utilizing two different techniques. These techniques included electrospinning with blending, in which the electrospinning solution and AuNPs were blended, and electrospinning/electrospraying, in which AuNPs were electrosprayed concurrently with electrospinning. A stabilization/carbonization technique was used to create the electrospun mats. In the electrospraying and mixing electrospinning modes, respectively, electrical conductivity increased by up to 29.2% and 81%. Indirect MTT and LDH toxicity assays were used to evaluate the toxicity of MG63 cells, but no discernible toxicity was found, and the scaffolds had no negative effects on cell growth. We can draw the conclusion that these scaffolds could be used in bone tissue engineering [92]. Table 1 summarizes the advantages and limitations of the above-mentioned nanofibers-based scaffolds prepared for bone tissue regeneration.

A critical step in the application of mineralized nanofibers is the characterization of the fabricated nanofibers. In this concept, the morphology of nanofibers (using SEM, TEM, and AFM microscopy), mechanical properties, swelling capacity, degradation state, hydrophilicity/hydrophobicity state, bioactivity/osteoactivity, hemocompatibility, cell viability/toxicity, immunogenicity, osteoconductivity, and osteoinductivity of the fabricated nanofibers must be evaluated.

## 7. Mineralization Process

### 7.1. Biomimetic Mineralization Approach

The biomimetic approach is considered as the mineralization process is performed in the aqueous solutions phase in ambient conditions or nearly ambient conditions using simulated body fluids (SBFs). These approaches are powerful methods that enable us to fabricate advanced mineralized nanofibers with complex shape, hierarchical organization, and controlled polymorph architecture and size under ambient conditions in aqueous environments. The biomimetic mineralization relies on the nucleation and crystal growth, where the crystals’ shape, size, aggregation, orientation, and texture can be controlled by thermodynamic or kinetic methods [95]. For complex structures to become mineralized, nucleation is the first and most important stage. The nuclei are usually unstable due to their high surface energy and grow soon after their formation, which makes this step difficult to study. It has been shown that the presence or induction of monodisperse nuclei on the nanofibers for the formation of nanocrystals with nearly monodisperse size while in the fast growth regime is critical for the formation of nanocrystals with the anisotropic shape.

**Figure 4:**
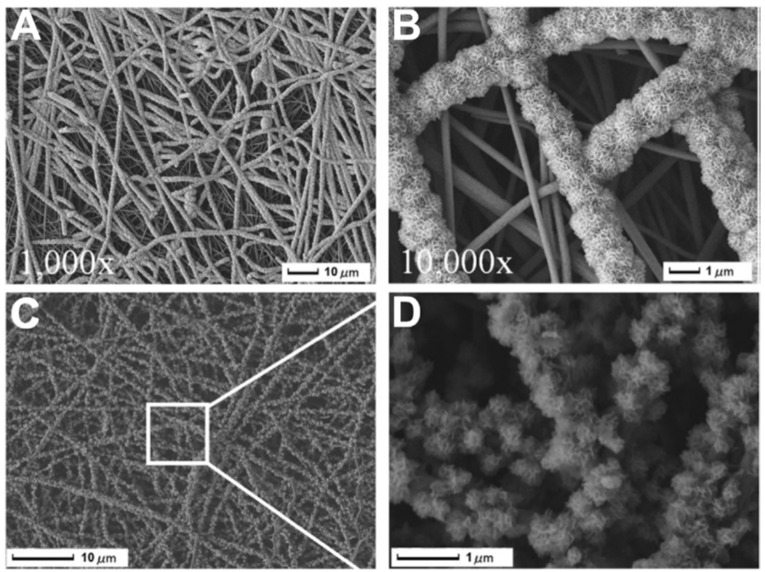
SEM photos show the development of HAp particles on T-CNF mats treated with NaOH aq. solution at concentrations of (**A**) 8%, (**B**) 16%, (**C**) 20%, and (**D**) the crystals that resemble flowers. One day for cultivation. Reproduced with permission from Ref. [97].

In the nucleation step, the ions of the mineralizing medium anchor on the nanofibers to form nuclei, and the presence or induction of proper surface functional groups determines the homogenous and monodisperse formation of the nuclei. In a previous study, we observed that the surface treatment of carbon nanofibers (CNFs) using concentrated NaOH and the formation of carboxyl groups resulted in the uniform and monodispersed formation of HA crystals [96]. In another study, Wu et al. [97] reported that the surface treatment and induction of carboxyl functional groups on CNFs not only induced the uniform mineralization but also the mineralization of fibers located at deeper parts (Figure 4).

### 7.2. Sequential Approach

**Figure 5:**
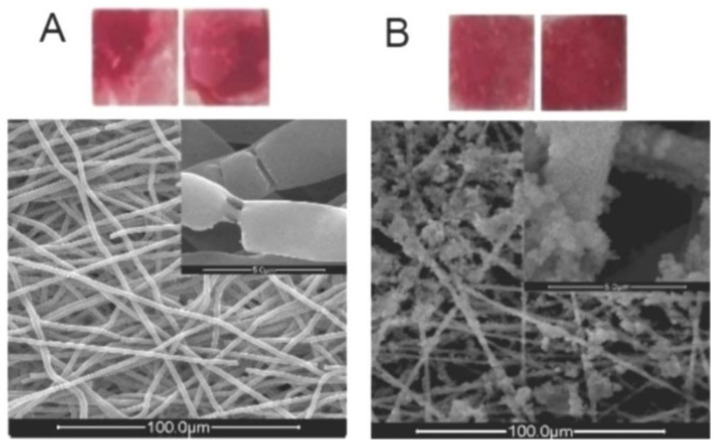
(**A**): Digital image of an ARS-stained sample that has been treated with the standard nucleation procedure (SNP) after being dipped in EtOH; bottom: SEM image of the same sample. (**B**): Digital image of an ARS-stained sample that has been treated with the SNP after being activated by ultrasonic waves; top: SEM image of the same sample. Reproduced with permission from Ref. [98].

The sequential mineralization approach is based on alternate dipping of the scaffold into the mineralization solutions containing calcium or phosphate ions. Using this method, it is possible to control the amount of mineralized phase formation, as well as the ratio of calcium/phosphate. Accordingly, the concentration of ions in the mineralization solution and the incubation time is determinant along with the surface properties of nanofibers. Several studies applied this technique to mineralize the nanofibrous scaffolds. The presence or induction of nucleation sites is critical for mineral phase deposition on the nanofibers. Luickx et al. [98] induced the sequential mineralization process to improve the osteoactivity of electrospun poly(D,L-lactide) (PLA) nanofibers. They used ethanol and demineralized water incubation along with ultrasonication to activate the surface of the nanofibers (Figure 5).

Then, the activated nanofibers were alternately immersed in the mineralization solutions (20 mL/cm^2^ 1000 mM CaCl_2_.2H_2_O and 20 mL/cm^2^ 600 mM Na_2_HPO_4_ solutions) and washed with water between the intervals. They reported that the nucleation step along with the mineralization parameters has significant effects on the physicochemical and biological properties of the final structure. The cell viability studies on MC3T3-E1 cells showed that the mineralization process significantly increased the viability of cells on the treated nanofibers compared with pristine nanofibers. Using the same approach, Luickx et al. [99] coated poly(e-caprolactone) nanofibers with a calcium phosphate layer to improve the hydrophilicity and biocompatibility of the nanofibers. The authors reported similar results and pointed out that the developed coating method can be applied for different nanofibers.

In another study, Kothapalli et al. [100] reported that the washing step parameters, such as the duration of washing and induction of agitation using the stirring process, also have determinant effects on the quantity of mineralization. In this study, they used 1N NaOH to activate the PLA nanofibers’ surface and induce –COO^-^ and -OH functional groups on the nanofibers as the nucleation sites. They also observed that increasing the soaking time as well as soaking repetitions increased the net amount of mineral phase deposition. The results showed that around 35 wt% of the mineral phase was deposited on the nanofibrous mat after six cycles of dipping. Itoh et al. [101] reported that the mineral phase deposition on chitosan tubes reached around 57 wt% after 15 cycles of soaking. Cui et al. [102] grafted chitosan on PLA nanofibers and applied the sequential mineralization approach to grow hydroxyapatite (HA) crystals on the nanofibers. According to their findings, the degree of chitosan grafting and the amount of HA development on the nanofibers both directly correlate with each other and depend on the aminolysis parameters (e.g., time and reagent). The in vitro studies showed that grafting and mineralization significantly improved the cytocompatibility of the scaffold.

### 7.3. Sol–Gel Approach

The combination of the electrospinning technique with the sol–gel approach is another fascinating method for fabricating mineralized nanofibrous scaffolds [103,104]. The conventional sol–gel process includes two different phases of solution (sol) and gelation (gel) [105,106]. For instance, the formation of a silica network using this approach follows a well-established three-stage process:

Hydrolysis: Si(OR)_4_ + nH_2_O → (OH)_n_Si(OR)_4−n_ + _n_ROH

Condensation: 2Si(OH)_4_ → (OH)_3_Si–O–Si(OH)_3_ + H_2_O

Gelation: Si(OR)_4_ + Si(OH)_4_ → (OH)_3_Si–O–Si(OR)_3_ + ROH

**Figure 6:**
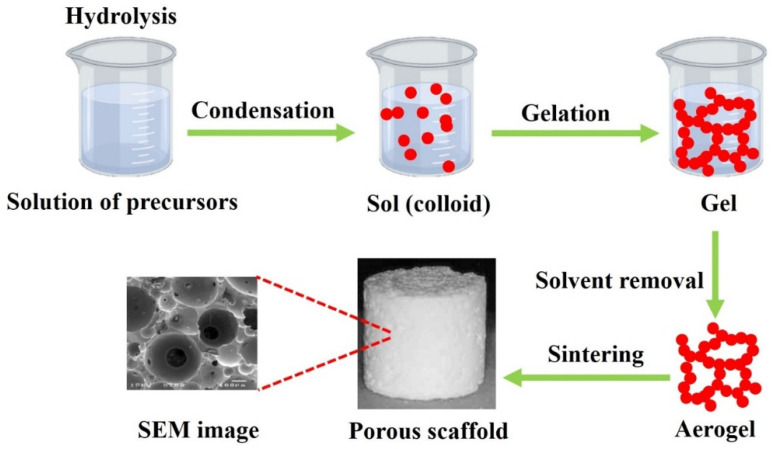
The conventional porous bioglass scaffold fabrication.

In this three-stage process, silanol groups form through hydrolysis of the silica precursor (usually tetraethyl orthosilicate (TEOS) and/or triethyl phosphate (TEP)) and condense through the silicon atoms’ cross-linking to form silica nanoparticles. This process is the basic principle of the sol–gel process that can be integrated into other approaches, such as particulate leaching and polymer polymerization, from 3D porous scaffolds (Figure 6).

**Figure 7:**
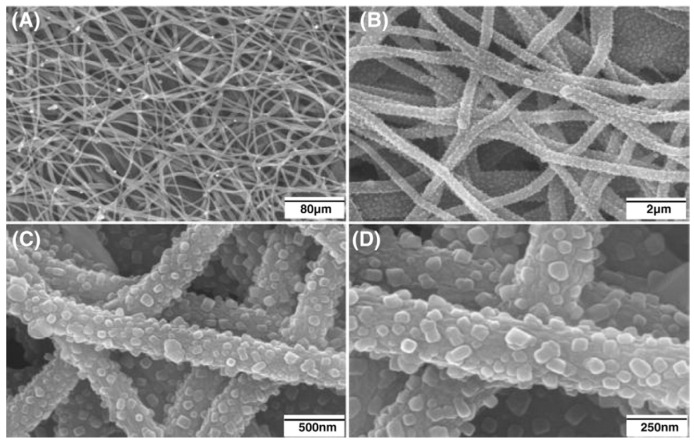
SEM photos of the distribution of well-dispersed β-TCP nanoparticles that firmly adhered to CNFs at various magnifications (**A**) 80 µm, (**B**) 2 µm, (**C**) 500 nm, (**D**) 250 nm. (Whereas the CNFs had an average diameter of about 300 nm, the average size of the β-TCP particles ranged from 30 to 60 nm). Reproduced with permission from Ref. [110].

Fabricating the mineralized electrospun nanofibers using the sol–gel method can be classified as the template-assisted sol–gel method that requires a precursor/carrying polymer. In this scenario, the precursor substances of the mineral phase are mixed with a carrier polymer (e.g., Polyvinylpyrrolidone (PVP) [107], Poly(vinyl alcohol) (PVA) [108], or Polyacrylonitrile (PAN) [109]) and converted to nanofibers. The calcination/sintering is the critical step to transform the fabricated nanofibers into mineralized nanofibers. The benefits of this method over the coating-based methods are the preservation of the original morphology and diameter of precursor nanofibers that provide a high surface-to-volume ratio, pore size, and porosity. These properties provide a higher adsorption of proteins, rapid dissolution of ions, controlled drug delivery, and higher osteogenic potential that subsequently result in a higher osseointegration and osteoconductivity. Liu et al. [110] blended the mineral phase precursor (triethylphosphate (TEP), calcium nitrate tetrahydrate) to the carrier polymer (PAN) and fabricated the electrospun precursor nanofibers. The created nanofibers were then carbonized in highly pure N_2_ and stabilized in air. The results showed that the formed mineral phase comprised calcium pyrophosphate (Ca_2_P_2_O_7_), HA, and β-tricalcium phosphate (β-TCP) crystals. The SEM images (Figure 7) clearly showed the transformation of the precursor nanofibers to mineralized nanofibers. The cell culture studies showed that the synthesized nanofibers supported the attachment and proliferation of MG-63 cells.

**Figure 8:**
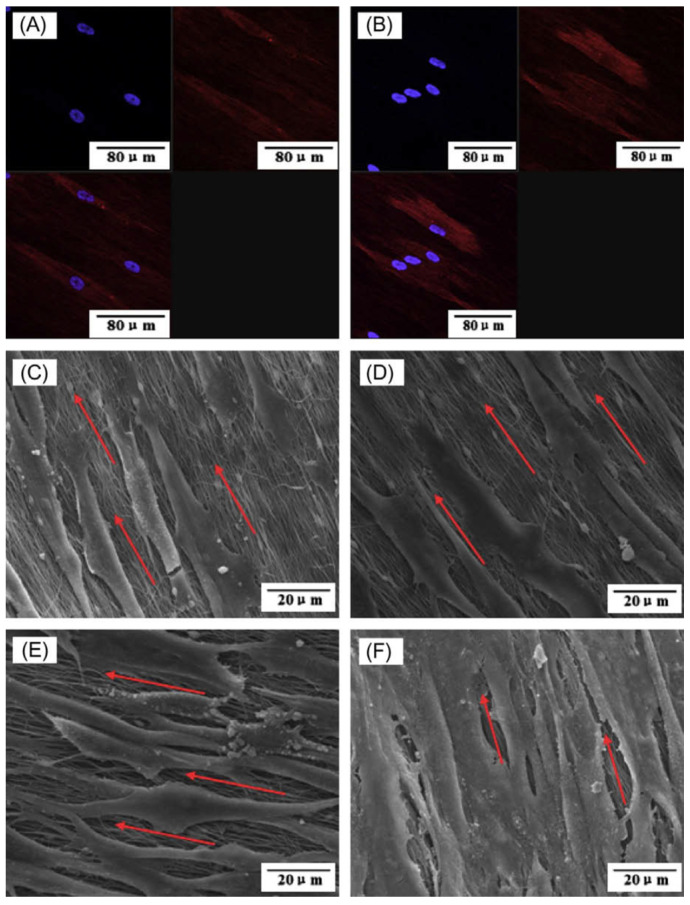
Morphology of PDLCs on the fabricated nanofibers. (**A**) CLSM image of cells on pristine CNFs, (**B**) CLSM image of cells on β-TCP-decorated CNFs, (**C**) SEM image of cells on pristine CNFs after 1 day of cell seeding, (**D**) SEM image of cells on pristine β-TCP-decorated CNFs after 1 day of cell seeding, (**E**) SEM image of cells on pristine CNFs after 7 days of cell seeding, and (**F**) SEM image of cells on pristine β-TCP-decorated CNFs after 7 days of cell seeding. Reproduced with permission from Ref. [112].

In another study, Han et al. [111] applied a similar method with slight modifications. They hydrolyzed the TEP solution before the incorporation into the polymeric solution. They also added TEOS to the precursor polymeric solution and applied the electrospinning. The results showed the formation of mineral crystals within and onto the nanofibers. The biological evaluations confirmed the bioactivity and biocompatibility of the fabricated nanofibers. Liu et al. [112] also combined the sol–gel mineralization process with the electrospun carbon nanofibers (CNFs) fabrication method. They used TEP as the phosphorus source and calcium nitrate tetrahydrate as the calcium source to fabricate β-TCP-decorated CNFs. The toxicity of the fabricated nanofibers against human periodontal ligament cells (PDLCs) revealed the biocompatibility of the nanofiber. The SEM and confocal laser scanning microscope (CLSM) imaging (Figure 8) showed that the cells grew on nanofibers along with the axis of aligned nanofibers.

## 8. Conclusions and Future Remarks

Bone tissue engineering is an alternative approach aiming to improve the healing outcomes of the conventional bone regeneration methods. In this scenario, the combination of nanomaterials, especially nanofibers, with bioceramic concepts can be promising to propose a sophisticated scaffold for bone tissue engineering. The current article reviews and discusses the application of mineralized nanofibers for bone tissue engineering applications, their structures, properties, and fabrication methods over the past few years. Despite their beneficial properties, mineralized nanofibers suffer from their 2D structure that limits translating them to the clinic. Alternatively, the combination of the mineralized nanofibers with 3D scaffolds as the nanocomposite can tailor them for clinical applications. Typical fabrication techniques for 3D scaffold preparations that have the potential to be combined with the mineralization process include rapid prototyping, hydrogels, thermal-induced phase separation, gas foaming, and particle leaching. From the authors’ point of view, the combination of an innovative and emerging manufacturing concept (electrospinning) with the partially long-lasting bone regeneration concept (bioceramics and mineralization) can provide scaffolds with the highest similarity to native bone structure and, subsequently, the highest healing/regeneration outcomes. However, more precise studies must be conducted to translate the proposed structure to the clinic.

## Figures and Tables

**Table 1:** Examples of some nanofibers-based scaffolds for bone tissue regeneration.

Scaffold Composition	Advantages	Limitations	Ref.
Hydroxyapatite (HA)-crystal-decorated osteoconductive electrospun CNFs	The size of the HA crystal (35.2 nm) is similar to that found in normal bone. The composites (24 M-CNFs) were biocompatible with negligible toxicity.	Pristine CNFs’ passive surface Pristine CNFs’ hydrophobic surface Low or non-biodegradability. The surface of the polymers needs to be functionalized.	[91]
Silica-nanoparticles-incorporated CNFs using electrospinning	The addition of silica NPs increased the hydrophilicity. Improved cell attachment, viability, and proliferation	Low flexibility of the resultant mat Non-biodegradation	[22]
CNF/gold nanoparticle (CNF/AuNP) conductive scaffolds	After being exposed to the furnace, the gold nanoparticles’ crystalline structure was unaltered. The composites were biocompatible.	Low or non-biodegradability. The surface of the polymers needs to be functionalized.	[92]
Electro-conductive electrospun CNFs-medicated DCF	Increased cell growth. Increased osteogenic activity.	Poor toughness Non-biodegradability. Hydrophobic surface Low processability.	[93]
Electro-conductive electrospun CNFs/Fe_2_O	Cytocompatible. Negligible toxicity (CNFs/Fe_2_O_3_ from PAN FeSO_4_.7H_2_O 15%)	Non-biodegradability. The surface of the polymers needs to be functionalized.	[94]

## Data Availability

Not applicable.
